# Supplementary material for: Spontaneous histone exchange between nucleosomes
Source: J Biol Chem. 2023 Jul 11;299(8):105037. doi: 10.1016/j.jbc.2023.105037 (PMC10406861; doi:10.1016/j.jbc.2023.105037)
Supplement: Supporting information [file mmc1.pdf]

Supporting Information

Spontaneous Histone Exchange Between Nucleosomes

Subhra Kanti Das, Mai Thao Huynh, and Tae-Hee Lee*

Department of Chemistry, The Pennsylvania State University, University Park, 16802, Pennsylvania, USA

*Corresponding author: txl18@psu.edu

Table S1. The sequences of DNA oligonucleotides used to construct the DNA mimetics for the two different H2A-H2B exchange cases. The labeling positions of Cy3, Cy5, and Cy5.5 are denoted in green, red, and brown, respectively. The mark iAmMC6T is for a Thymine analog with an amine terminated C6 linker.

Distal Case	
S1	/5 biosg/ ACGCGAGC/iAmMC6T/CAGGAGCA
S2	/5Cy5/TGCTCCTGAGCTCGCGT/3Cy55Sp/
Proximal Case	
S3	/5 biosg/ ACGCGAGC/iAmMC6T/CAGGAGCA
S4	/5Cy55/TGCTCCTGAGCTCGCGT/3Cy3Sp/

Table S2. The fitting results from the charts shown in figure 3. The apparent kinetic rate constants are in h^{-1} and not corrected for the nucleosome concentration.

	**4 °C, 10 mM NaCl (h^{-1})**	**4 °C, 50 mM NaCl + 150 mM KCl (h^{-1})**	**25° C, 10 mM NaCl (h^{-1})**	**+ Nap1, 4°C (h^{-1})**
**Unmodified**	0.513 ± 0.092	0.989 ± 0.158	0.595 ± 0.090	0.982 ± 0.137
**CpG methylated**	0.396 ± 0.059	0.570 ± 0.072	0.574 ± 0.077	0.901 ± 0.104
**H3K56ac**	1.44 ± 0.28	1.32 ± 0.20	1.50 ± 0.26	1.97 ± 0.24

Table S3. The sequences of DNA oligonucleotides used to construct the nucleosomal DNA. For Cy3/Cy5 labeling along the DNA, the color-marked modified nucleotides in red and green, respectively for Cy3 and Cy5, were utilized. iAmMC6T is a Thymine analog with an amine terminated C6 linker is attached.

F1	34	/5Biosg/GCAG ATCGAGAATC CCGGTGCCGA GGCCGCTCAA
F2	77	/5Phos/ TTGG /iAmMC6T/CGTAGACAG CTCTAGCACC GCTTAAACGC ACGTACGCGC TGTCCCCCGC GTTTTAACCG CCAAGGGGAT TAC
F3	40	/5Phos/ TCC C/iAmMC6T/AGTCTCCA GGCACGTGTC AGATATATAC ATCCGAT
R1	69	ATCGGATGTA TATATCTGAC ACGTGCCTGG AGACTAGGGA GTAATCCCCT TGGCGGTTAA AACGCGGGG
R2	78	/5Phos/G ACAGCGCGTA CGTGCGTTTA AGCGGTGCTA GAGCTGTCTA CGACCAATTG AGCGGCCTCG GCACCGGGAT TCTCGAT

Figure S1. Control experiments to confirm fluorescence intensity signatures from H2A-H2B exchanged nucleosomes. (A) The structure of a nucleosome core particle with the labeling positions marked (left). The estimated distances between the fluorophores are shown (right). (B) The structures of 15 bp biotinylated dsDNA labeled with fluorophores at the marked positions that can mimic the H2A-H2B exchanged nucleosomes according to the distances estimated in A. (C) Intensity traces from fluorophores labeled at DNA mimetics are shown in B. In case of the proximal H2A-H2B exchange mimetic (left), a high level of Cy5.5 and a low level of Cy3 and Cy5 signals were observed, followed by photobleaching. In case of the distal H2A-H2B exchange mimic (right), a low level of Cy3 and a mid-high level of Cy5 and Cy5.5 signals were observed.

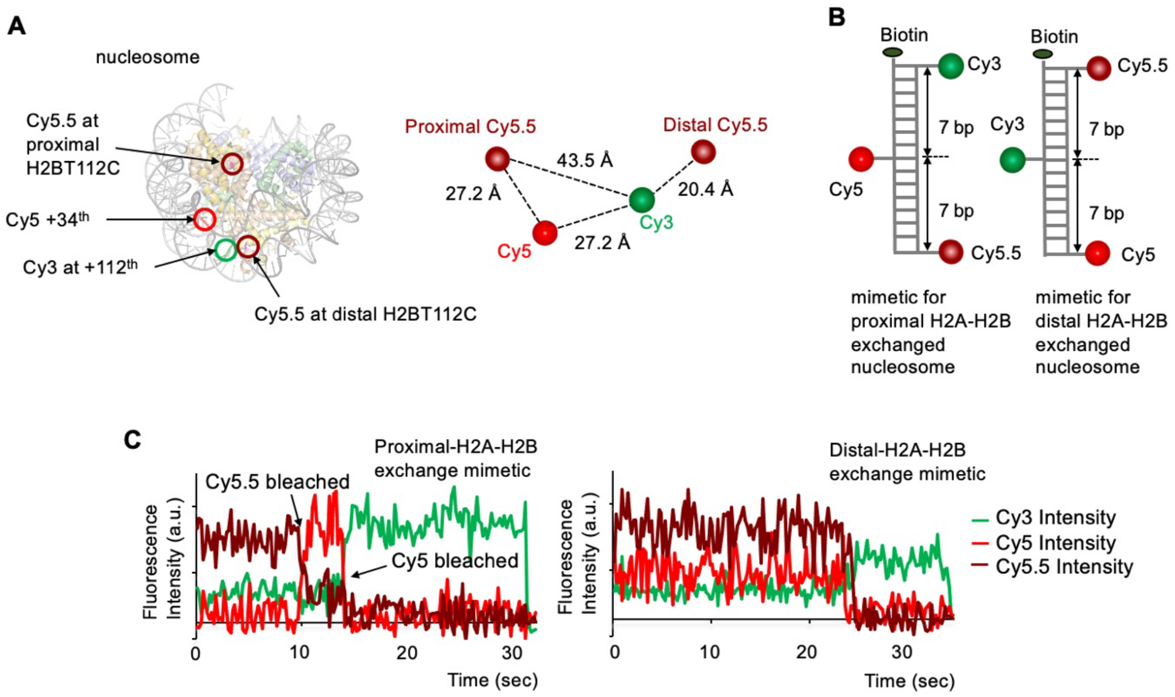

Figure S2. Histograms of FRET$_{Cy5}$ and FRET$_{Cy5.5}$ at 0 and 18th hour time points from the nucleosomes showing zero intensities after photobleaching (single nucleosomes for accurate background correction), longer photobleaching lifetimes (> 10 sec), and a decent signal-to-noise ratio (> 4) for (A) unmodified, (B) histone H3K56 acetylated (0 and 8th hour), and (C) CpG methylated nucleosomes. It is evident in all cases that both non-zero FRETCy$_{5.5}$ and lower FRET$_{Cy5}$ grow as time passes (see red-circled areas), indicating that H2A-H2B-Cy5.5 is being incorporated into the nucleosomes labeled with a Cy3-Cy5 pair. Note that the histogram counts cannot be used as a measure of the percent exchange as Cy5.5 photobleaches much faster than Cy5. It is typical according to our experience that fluorophores labeled on protein photobleach much faster than those labeled on nucleic acids.

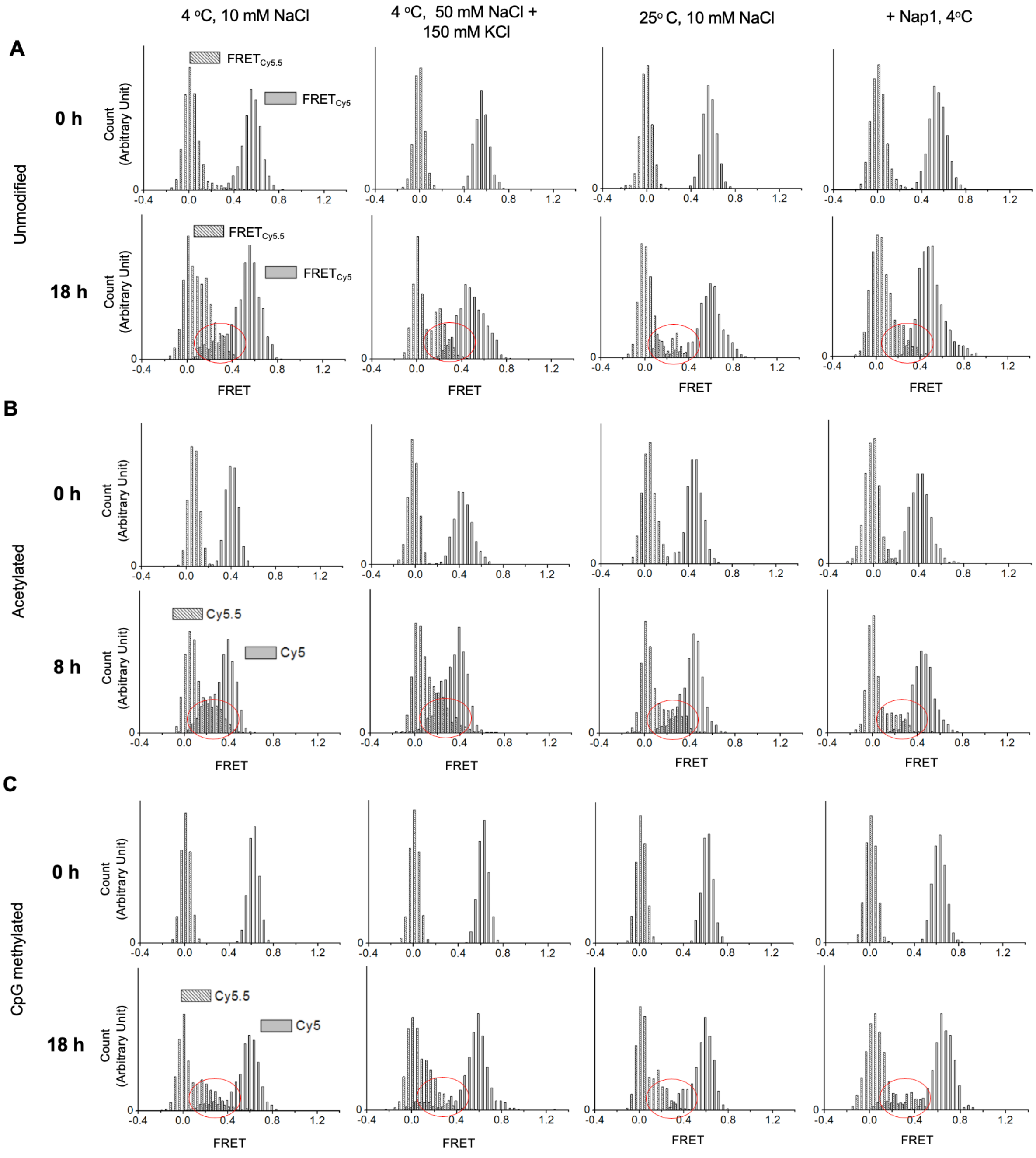

Figure S3. A mass-spectrometric analysis result confirms the modification of histone H3 to introduce H3K$_S$56ac (Mw = 15297.6, expected Mw = 15298). The areas under the peaks suggest that 90% of H3K56 were sucessfully acetylated. This is the same histone H3 as was used in a previous publication (Huynh et al., *ACS Chem Biol* 2020 15 3133). Copyright 2020 American Chemical Society.

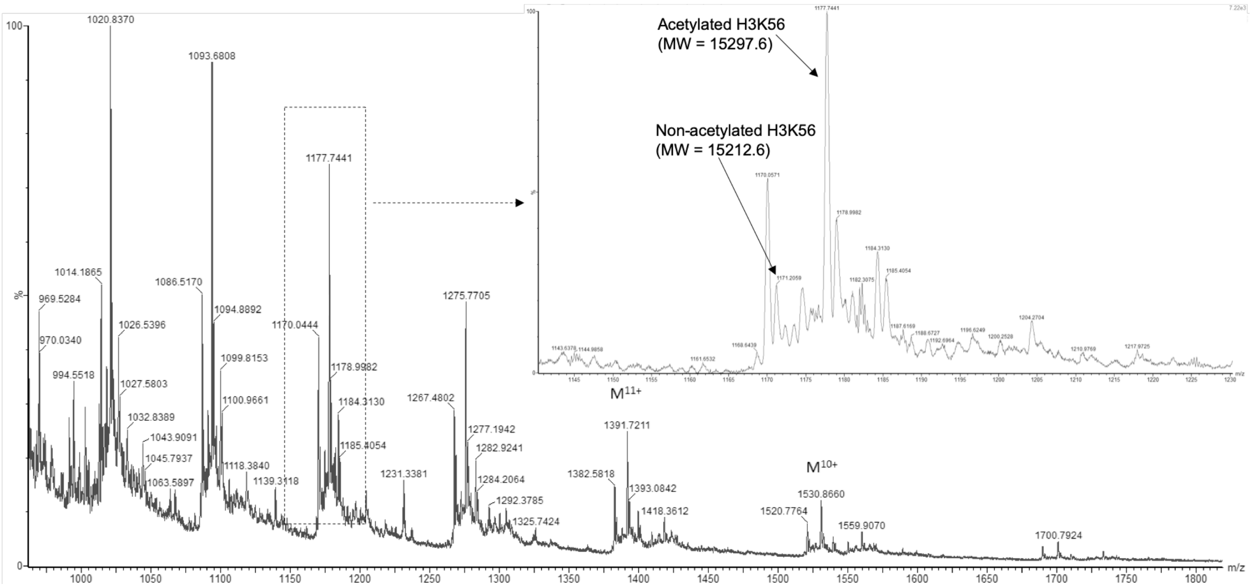

Figure S4. Successful CpG methylation was confirmed with a DNA digestion assay. The PAGE gel shown below confirms that the activity of the BstUI restriction enzyme was blocked at the two target CGCG sites (marked red below) of the CpG-methylated 601 DNA sample while it digested the vast majority of the unmethylated DNA. This enzyme activity is known to be blocked when the target DNA site (CGCG) is CpG methylated (McClelland et al., *Nucleic Acids Res* (1981) 9 5859). The digestion of the unmethylated DNA should produce three fragments of 71, 12, and 64 bp (cleavage sites are marked with "/" below). The two bands between 50 and 100 bp confirm the 71 and 64 bp fragments. The 12 bp band is likely too weak to produce a noticeable mark on the gel under the imaging conditions. The gel image was taken with the automatic exposure function in a ChemiDoc^{TM} XRS+ system with an Image Lab^{TM} software package (Bio-Rad Laboratories Inc., Hercules CA) and has not been altered in any way.

Digestion conditions: 10 minutes incubation at 60 °C of a 50 µL reaction mixture (1 µg DNA and 10 units of the BstUI enzyme (New England Biolabs) in 20 mM Tris-OAc (pH 7.9), 10 mM $Mg(OAc)_2$, 50 mM KOAc, and 100 µg/mL recombinant albumin) followed by quenching and purification with a QiAquick PCR purification kit (QIAGEN).

601 DNA sequence and BstUI restriction sites: ATCGAGAATCCCGGTGCCGAGG CCGCTCAATTGGTCGTAGACAGCTCTAGCACCGCTTAAACGCACGTA**CG**/**CG**CTGTC CCC**CG**/**CG**TTTTAACCGCCAAGGGGATTACTCCCTAGTCTCCAGGCACGTGTCAGAT ATATACATCCGAT

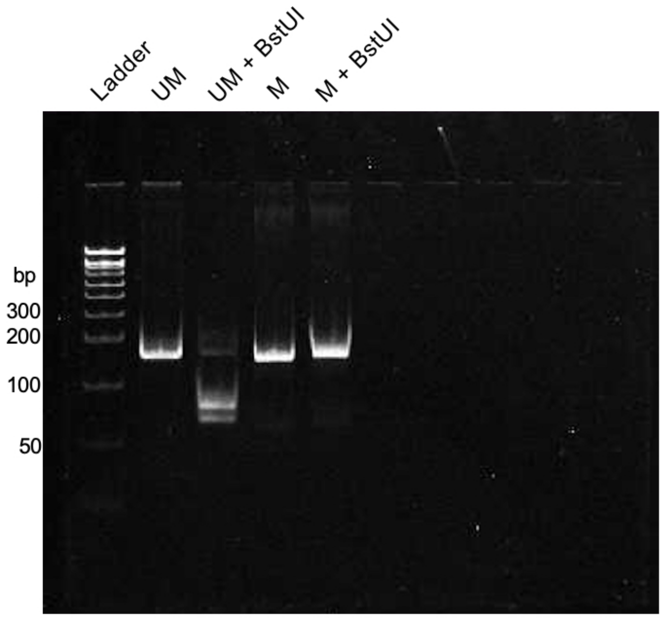

UM: Unmethylated DNA
UM + BstUI: Unmethylated DNA incubated with BstUI
M: Methylated DNA
M + BstUI: Methylated DNA incubated with BstUI

Figure S5. SDS-PAGE gels confirm properly purified histone octamers and dimers/teteramers. For the assembly of nucleosomes with H3K$_S$56ac (Cy5.5-labeled and -unlabeled), we used H2A-H2B dimers and (H3-H4)$_2$ tetramers separately. For these nucleosomes, the SDS-PAGE gel shown on the right confirms nucleosomes assembled around a histone octamer core. (Left) An SDS-PAGE gel shows properly assembled unmodified *X. laevis* histone octamer (lane #2, WT histone octamer, purchased from the Histone Source, Colodaro State University) and Cy5.5-labeled *X. laevis* histone octamer (lane #3, Cy5.5 histone octamer). (Right) An SDS-PAGE gel shows the unmodified histone octamer (lane #1, WT histone octamer, purchased from the Histone Source, Colodaro State University) and the core histones of nucleosomes assembled with *X. laevis* histone octamer containing H3K$_S$56ac (lane #2, H3K$_S$56ac nuc) and Cy5.5-labeled *X. laevis* histone octamer containing H3K$_S$56ac (lane #3, H3K$_S$56ac+Cy5.5 nuc).

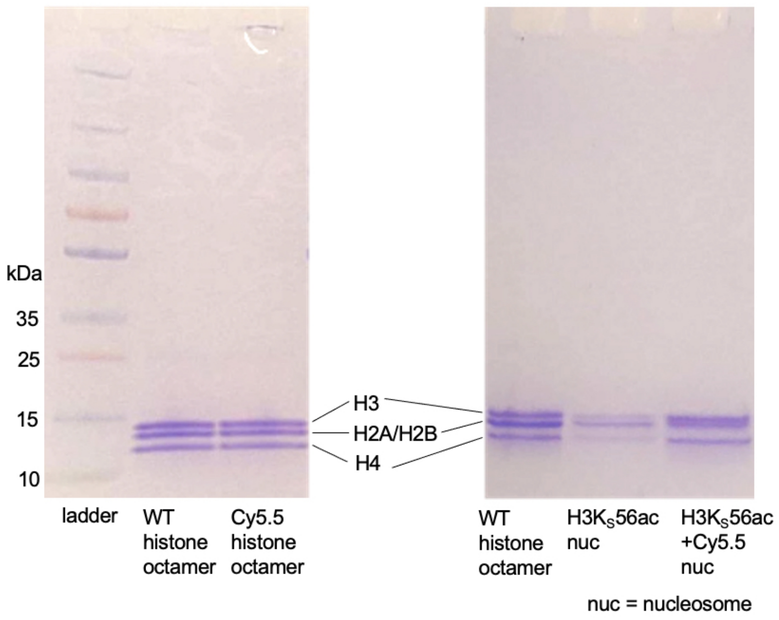

Figure S6. Native PAGE analyses for nucleosomal DNA and nucleosomes. (A) The left two lanes (L_{DNA} and U_{DNA}) show Cy3/Cy5 labeled and unlabeled nucleosomal DNA, respectively, and the right two lanes (L_{Ac} and U_{Ac}) show H3 K56 acetylated nucleosomes with labeled and unlabeled DNA, respectively. (B) Lanes L_{un} and U_{un} show unmodified nucleosomes with labeled and unlabeled DNA. Lanes L_{MT} and U_{MT} show CpG methylated nucleosomes with labeled and unlabeled DNA. Note that the nucleosomes with unlabeled DNA have their H2B labeled with Cy5.5.

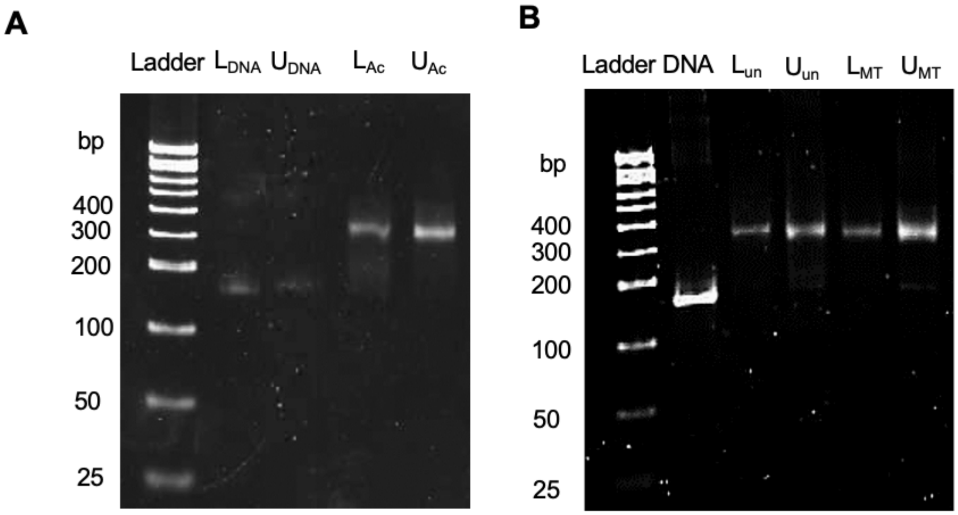

Figure S7. Nucleosomes with different labeling possibilities during the histone exchange reaction. (A) A total of eight different nucleosome species with a different fluorophore labeling scheme can exist in the reaction mix. (B) The full list of possible combinations of histone exchange between the eight species shown in A.

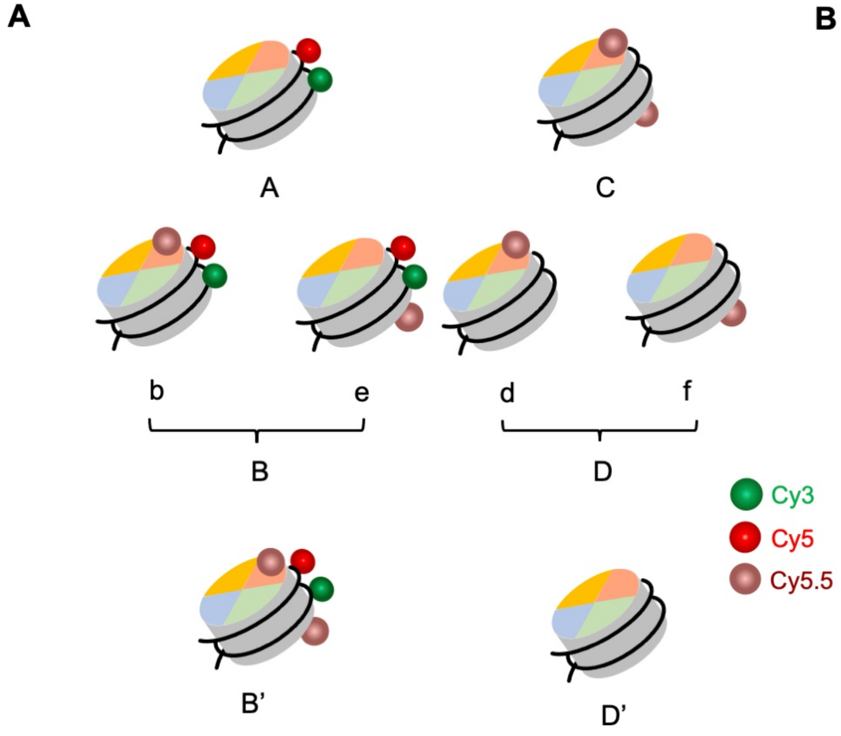

B

A+C	→	B+D
A+D	→	B+D’
	→	A+D
A+D’	→	A+D’
B+C	→	B+C
	→	B’+D
B+D	→	2(B+D)
	→	B’+D’
	→	A+C
B+D’	→	B+D’
	→	A+D
A+B	→	2(A+B)
C+D	→	2(C+D)
C+D’	→	D+D
D+D’	→	D+D’
A+A	→	A+A
B+B	→	B+B
	→	A+B’
C+C	→	C+C
D+D	→	D+D
	→	D’+C
D’+D’	→	D’+ D’
